# Three leading suicide methods in the United States, 2017–2019: Associations with decedents' demographic and clinical characteristics

**DOI:** 10.3389/fpubh.2022.955008

**Published:** 2022-11-17

**Authors:** Namkee G. Choi, C. Nathan Marti, Bryan Y. Choi

**Affiliations:** ^1^Steve Hicks School of Social Work, University of Texas at Austin, Austin, TX, United States; ^2^Department of Emergency Medicine, Philadelphia College of Osteopathic Medicine and BayHealth, Dover, DE, United States

**Keywords:** suicide methods, firearms, hanging/suffocation, poisoning, mental disorders, physical health problems, financial stress

## Abstract

**Background and aims:**

The U.S. suicide data show that use of lethal methods, specifically firearms and hanging/suffocation, has been increasing among young and middle-aged adults of both sexes over the past decades. In this study, we examined demographic and clinical correlates of use of firearms, hanging/suffocation, and poisoning as suicide methods among suicide decedents age 18+.

**Methods:**

Data came from the 2017-2019 National Violent Death Reporting System (NVDRS; *N* = 94,454, age ≥ 18 at the time of suicide; 74,042 men and 20,412 women). We fit generalized linear models (GLMs) for a Poisson distribution with a log link to examine (1) changes in four suicide methods (firearms, hanging/suffocation, poisoning, and other) during the study period, controlling for sex and age group; and (2) correlates of firearms, hanging/suffocation, and poisoning use.

**Results:**

In all age groups, 55% of men and 30% of women used firearms; 28% of men and 29% of women hanging/suffocation; 9% of men and 32% of women poisoning, and 8% of men and 9% of women “other” methods. Men age < 45 had higher likelihood of firearm and/or hanging/suffocation use than those age 45–64. Women age<45 also had higher likelihood of hanging/suffocation than those age 45–64. Prior suicide attempt history was associated with higher likelihood of poisoning in both sexes and hanging/suffocation in men; mental disorders/SUD were associated with higher likelihood of hanging/suffocation and poisoning in both sexes; physical health problems were associated with higher likelihood poisoning in both sexes and firearm use in men; relationship problems were associated with higher likelihood of firearm use; legal problems and job/financial/housing problems were associated with higher likelihood of hanging/suffocation in both sexes; and more crises were associated with higher likelihood of firearm use in both sexes.

**Implications:**

The findings call for the following suicide prevention strategies: (1) restricted access to firearms; (2) improved access to mental health/substance use treatment; (3) improved long-term and palliative care services for those (mostly older adults) with physical health problems; (4) financial/housing support policies to mitigate economic hardship; and (5) more research to identify effective strategies to curtail the increasing use of firearm and hanging/suffocation among young and middle-aged adults.

## Introduction

The overall suicide rates in the U.S. increased 30% between 2000 and 2020 (1). Although rates declined in 2019 and 2020 compared to 2018 in most age groups, suicide was the cause for 46,000 deaths, or one death every 11 min, in 2020 (1). Firearms (including handguns, rifles, shotguns or other large firearms, or other unspecified firearms) have consistently been used more frequently than any other methods among men, and male firearm suicides increased steadily since the mid-2000's to reach an age-adjusted rate of 12.5 per 100,000 population in 2020 (1). Firearms have also been the leading method of suicide for rural females (2), but use of firearms among women in general also increased significantly since 2008, and they became the leading method for all female decedents in 2020 (1.9 per 100,000) (1).

Following a steady increase over the past two decades, hanging/suffocation (including strangulation or other methods resulting in oxygen deprivation) was the second most frequently used method in both sexes in 2020 (6.1 for men and 1.7 for women per 100,000) (1). Poisoning (overdose of medicinal and nonmedicinal substances such as gases or other toxic materials) has been declining since mid-2000's in both sexes and was the third leading method for both sexes in 2020 (1.7 for men and 1.5 for women per 100,000) (1).

Firearms and hanging/suffocation as suicide methods, with case fatality rates (CFRs) of 89.7% and 84.5%, respectively, are far more lethal than poisoning (CFR = 8.0% for drug/liquid poisoning and 56.6% for gas poisoning) (3). Method-specific CFRs are higher for men than women and older persons (4). However, suicide mortality data show that increased rates of firearm and hanging/suffocation suicides are largely due to young and middle-aged adults. A study based on the 2006–2015 Nationwide Inpatient and Emergency Department Sample found that along with increases in incidence rates of total suicidal acts, use of more lethal methods (i.e., those with higher CFRs) among the 20–44 age group since 2009 and among the 45–64 age group since 2012 contributed to increasing suicide mortality rates (5). Recent Centers for Disease Control and Prevention (CDC) data also show that the increased firearm suicide rates were largely due to significant increases of firearm use among individuals age under 45, as consistently high rates of firearm use among older adults, older men in particular, have remained largely the same (6).

With increasing use of firearms and hanging/suffocation as suicide methods among women and younger age groups, more research is needed to examine other demographic and clinical characteristics associated with the choice of suicide methods. Identification of these characteristics may help better target individuals at high risk of suicide and reduce their access to lethal methods. Previous studies (7–10), all based on National Violent Death Reporting System (NVDRS) data, mostly examined characteristics of decedents who used firearms. However, since NVDRS in earlier years included a limited number of participating states, updated research with more nationally representative data is needed. More importantly, despite steadily increasing use of hanging/suffocation in both sexes, little research has been done on the characteristics of people who died by hanging/suffocation. Research is also needed to examine those who died by poisoning.

In this study based on the 2017-2019 NVDRS, we first examined sex and age group differences in suicide methods (firearms, hanging/suffocation, poisoning, and other) among suicide decedents age 18+ and changes in the use of these methods during the three-year study period. We then examined demographic and clinical correlates of use of firearms, hanging/suffocation, and poisoning in male and female decedents. Based on previous study findings (9, 10), our exploratory hypotheses are that in both sexes, older age, region of residence other than Northeast, physical health problems, and relationship problems would be associated with higher likelihood of firearm use, whereas previous suicide attempt and psychiatric and substance use problems would be associated with higher likelihood of poisoning. Studies have shown that compared to the Northeastern region, firearm suicides are more common in states in the Western, Midwestern, and Southern regions where firearm ownership rates are higher (9, 11). While research on hanging/suffocation in other counties has been extensive (12, 13), there is a paucity of research on hanging/suffocation in the U.S. Thus, we did not posit any hypothesis regarding its clinical correlates; however, based on the increasing use of hanging/suffocation among younger age groups, we hypothesized that those age under 45 would be more likely to have used this method. The findings will provide insights into the demographic and clinical characteristics of suicide decedents who used different methods of injury.

## Materials and methods

### Data source

We focused on suicide decedents aged 18 and older in the 2017–2019 NVDRS (*N* = 94,454, ages 18–105 at the time of death; 74,042 men [78.4%] and 20,412 women [21.6%], after excluding 3 decedents with missing data on sex). NVDRS is the only state-based violent death reporting system in the US that provides information and context on when, where, and how violent deaths occur and who is affected (14). NVDRS links data from death certificates and reports from coroners/medical examiners (CME) and law enforcement (LE) agencies on cases of violent deaths–suicides, homicides, deaths from legal intervention (i.e., victim killed by LE acting in the line of duty), deaths of undetermined intent, and unintentional firearm deaths. CME/LE reports are from the injury/death scene, ongoing investigations, accounts from family/friend and informants, and suicide notes, if available, and often serve as the basis of the circumstances of death as well as NVDRS variables that were “calculated” (coded “Yes” when endorsed by the CME and/or LE reports vs. “No/not available/unknown”). When available, crime lab and toxicology reports included in CME reports are also abstracted and entered in NVDRS.

We used 2017-2019 NVDRS data because the number of participating states increased from 27 in 2017 to 37 in 2017 and to 43 states, the District of Columbia, and Puerto Rico in 2019, although not all states provided complete data for all 3 years (15). Our preliminary analysis showed that some important results vary depending on the number of participating states. The authors of this study were granted access to de-identified NVDRS data for this study by the CDC's NVDRS-Restricted Access Data (RAD) review committee. This study based on de-identified data on decedents was exempt from the authors' institutional review board's review.

### Measures

#### Suicide methods

These were identified from the International Classification of Diseases, 10^th^ Revision (ICD-10), codes for intentional self-harm (X60-X84) for underlying cause of death in death certificates and/or from the underlying cause descriptions in CME reports. They included the following: firearms; hanging/suffocation; poisoning due to any type of alcohol/drug/medicine/chemical overdose or with gas (e.g., carbon monoxide, nitrogen); laceration/sharp instruments; blunt objects; jumping from heights; contact with moving objects (train/other vehicles); drowning; and other (fire, hypothermia, electrocution, starvation, dehydration, not adhering to or refusing medical care, other specified but not elsewhere classified methods, and unspecified methods). We classified them into four categories in this study: firearms, hanging/suffocation, poisoning, and all other methods.

#### Demographic variables

Data on age at the time of death, sex, race/ethnicity, level of education, military service history, and US Census region of residence were from the death certificates and CME/LE reports. In this study, decedents' ages were categorized into 18–24, 25–44, 45–64, and 65+ years to examine age group differences, and the 45–64 age group was used as the reference category in multivariable models given that the largest numbers of decedents in both sexes belonged to the age group.

#### Suicide attempt history and intent disclosure

These were based on CME/LE reports. History of suicide attempts refers to any previous suicide attempt before the fatal incident, regardless of the severity and injury status. Intent disclosure refers to: (a) disclosure of suicidal thoughts or intent to die by suicide to another person *via* verbal, written, or electronic communications within a month (or recently) before suicide (i.e., not at the moment of the suicide), whether explicitly (e.g., “I plan to go to my cabin with my gun and never come back”) or indirectly (e.g., “I know how to put a permanent end to this pain”), or (b) a separate suicide attempt within a month of the suicide. We examined the differences in leaving a suicide note by suicide methods for descriptive purposes only.

#### Mental health and substance use problems

In NVDRS, mental health and substance misuse problems, based on CME/LE reports, were recorded “Yes” without the need for any indication that they directly contributed to the death and included the following: (1) depressed mood at the time of death (without the need for a clinical diagnosis); and (2) any diagnosed mental health problem [disorders and syndromes listed in DSM-5 (16)] at the time of death; (3) alcohol problem/addiction; and (4) other substance misuse/addiction (e.g., prescription drug misuse, chronic/abusive/problematic marijuana use, any use of other illicit drugs or inhalants). Additionally, we included any other addiction (e.g., gambling, sex) that appears to have contributed to the death. Based on data on mental disorders, substance misuse, and other addiction, we created a summary variable with the following four categories: No mental disorder or substance misuse/addiction (referred to as SUD [substance use disorder] hereafter); SUD only; mental disorder only; and both mental disorder and SUD. We also reported any history of mental health/substance use treatment for descriptive purposes only (as the data on treatment status are likely to be incomplete as they were reported by family/friends/other informants, not from healthcare professionals or official medical records [email communication with the NVDRS-RAD team; April 19, 2022]).

#### Physical health problems and relationship and other life stressors as suicide precipitants

These were based on CME/LE reports. Physical health problem was recorded “Yes” only if any diagnosed or perceived physical health problem (e.g., terminal disease, debilitating condition, chronic pain) was relevant to the death (e.g., “despondent over recent diagnosis of cancer” or “complained that he could not live with the pain associated with a condition” even if the condition may not have been diagnosed or existed).

Relationship/other life stressors included: (1) relationship problems (conflict with an intimate partner and/or other family members, arguments, other family stressors, caregiver burden, or abuse by a caregiver); (2) recent suicides or other deaths of family/friends or a traumatic anniversary; (3) job/finance/housing problems; and (4) criminal/civil legal problems.

#### Number of crises

NVDRS provides a variable that is the count of crises (“current/acute event within 2 weeks of death”) that the decedent faced with respect to mental disorder, SUD, physical health, and relationship and other life stressors discussed above.

### Analysis

All statistical analyses were performed using Stata/MP 17. First, we used cross-tabulations with Pearson's χ^2^ tests to describe suicide methods by sex and age group. Second, we fit four generalized linear models (GLMs) for a Poisson distribution with a log link to examine changes in four suicide methods (firearms, hanging/suffocation, poisoning, and other) during the study period, controlling for sex and age group. Third, we used cross-tabulations with Pearson's χ^2^ tests and one-way ANOVA to compare demographic and clinical characteristics of decedents by their suicide methods separately for men and women. Finally, we tested the study hypotheses regarding correlates of suicide methods (firearms, hanging/suffocation, and poisoning, separately for men and women) with six GLMs for a Poisson distribution with a log link. Since our aim was to examine correlates of firearm vs. no firearm use, hanging/suffocation vs. no hanging/suffocation, and poisoning vs. no poisoning, each method (e.g., firearm use vs. nonuse) served as the dependent variable. We did not fit the multivariable model for “other” methods as that category included many different methods, which precludes clearly interpretable results. We fit GLMs rather than logistic regression models as odds ratios exaggerate true relative risk to some degree when the event (i.e., the specific method used) is a common (i.e., >10%) occurrence (17). The independent variables for all six GLM models were demographics, contributing physical health problems, mental disorder/SUD, relationship/other life stressors, and other clinical characteristics. As a preliminary diagnostic, we used variance inflation factor (VIF), using a cut-off of 2.50 (18), from linear regression models to assess multicollinearity among covariates. VIF diagnostics indicated that multicollinearity was not a concern. GLM results are reported as incidence rate ratios (IRRs) with 95% confidence intervals (CIs). Significance was set at p < 0.05.

## Results

### Suicide methods by sex and age group and changes from 2017 to 2019

Table 1 shows that age group distributions of decedents in both sexes. Firearms were used by 54.7% of all male decedents and 30.0% of all female decedents; hanging/suffocation by 28.4% of all male decedents and 28.5% of all female decedents; poisoning by 9.0% of all male decedents and 32.1% of all female decedents; and other methods by 7.0% of all male decedents and 9.4% of all female decedents.

**Table 1:** Suicide methods by sex and age group of suicide decedents, 2017-2019 (column %).

***N* (% of sex)**	**Men (*****N*** = **70,042; 78.4% of both sexes)**					**Women (*****N*** = **20,412; 21.6% of both sexes)**				
	**All**	**18–24 years *N* = 8,388 (11.3)**	**25–44 years *N* = 25,158 (34.0)**	**45–64 years *N* = 25,640 (34.6)**	**65+ years *N* = 14,856 (20.1)**	**All**	**18–24 years *N* = 1,859 (9.1)**	**25–44 years *N* = 6,924 (33.9)**	**45–64 years *N* = 8.568 (42.0)**	**65+ years *N* = 3,061 (15.0)**
Firearms	54.7	52.6	45.6	52.5	75.2	30.0	26.9	28.0	30.9	33.7
Hanging/suffocation	28.4	32.7	38.1	27.3	11.6	28.5	43.8	37.4	22.6	15.9
Poisoning	9.0	5.8	8.1	11.8	7.6	32.1	19.4	25.4	37.1	40.8
Other^a^	7.9	8.9	8.2	8.4	5.6	9.4	9.9	9.2	9.4	9.6
Age group difference in methods: *P*-value (Pearson χ^2^)		Firearms: *p* < 0.001; Hanging/suffocation: *p* < 0.001; Poisoning: *p* < 0.001; Other: *p* < 0.001					Firearms: *p* < 0.001; Hanging/suffocation: *p* < 0.001; Poisoning: *p* < 0.001; Other: *p* = 0.851			

a Jump from a high place, blunt force from moving vehicle/train/other, sharp or blunt object, drowning, smoke/fire/flame/electrocution/hypothermia, not adhering to or refusing medical care, other specified not elsewhere classified method, and unspecified methods.

With respect to age group differences among men, firearm use was most common in the 65+-age group (75.2%), followed by the 18–24, 45–64 years, and 25–44 age groups; hanging/suffocation was most common in the 25–44 age group (38.1%), followed by the 18–24, 45–64, and 65+ age groups; and poisoning was highest in the 45–64 age group (11.8%), followed by the 25–44, 65+, and 18–24 age groups. Among women, firearm use was highest in the 65+ age group (33.7%), followed by the 45–64, 25–44, and 18–24 age groups; hanging/suffocation was most common in the 18–24 age group (43.8%), followed by the 25–44, 45–64, and 65+ age groups; and poisoning was highest (40.8%) in the 65+ age group, followed by the 45–64, 25–44, and 18–24 age groups.

Table 2 shows that compared to 2017, while controlling for sex and age group, firearm suicides increased 3% in 2019 and poisoning suicides decreased 6% in 2018 and 7% in 2019. Changes in hanging/suffocation were not significant. Without adjusting for other demographic and clinical characteristics, men's risk of firearm use was 80% higher but risk of poisoning was 71% lower than women's. Sex difference in hanging/suffocation was not significant. Compared to the 45–64 age group, the 65+ age group's risk for firearm use was 40% higher, but the 25–44 age group's risk was 13% lower; however, the 65+ age group's risk for hanging/suffocation was 53% lower, but the 18–24 and 25–44 age groups' risks were 30% and 50% higher, respectively. Compared to the 45–64 age group, the other three age groups were 16–49% less likely to have used poisoning. Men and those age 65+ (compared to those age 45–64) were less likely to have used other methods.

**Table 2:** Changes in suicide methods, 2017-2019, controlling for age and sex: Results from generalized linear models for a Poisson distribution with a log link.

	**Firearm**	**Hanging/suffocation**	**Poisoning**	**All other methods**
	**IRR (95% CI)**	**IRR (95% CI)**	**IRR (95% CI)**	**IRR (95% CI)**
Year: vs. 2017				
2018	1.01 (0.99–1.04)	1.01 (0.98–1.04)	0.94 (0.90–0.98)^**^	1.01 (0.95–1.06)
2019	1.03 (1.01–1.05)^*^	1.00 (0.97–1.03)	0.93 (0.89–0.97)^**^	0.96 (0.91–1.02)
Male vs. Female	1.79 (1.74–1.84)^***^	1.01 (0.98–1.04)	0.29 (0.28–0.30)^***^	0.84 (0.80–0.89)^***^
Age group: vs. 45–64 years				
18–24 years	0.98 (0.95–1.02)	1.33 (1.28–1.38)^***^	0.51 (0.48–0.55) ^***^	1.06 (0.99–1.14)
25–44 years	0.87 (0.85–0.89)^***^	1.45 (1.41–1.49)^***^	0.69 (0.66–0.72)^***^	0.99 (0.94–1.04)
65+ years	1.39 (1.36–1.42)^***^	0.47 (0.45–0.49)^***^	0.84 (0.80–0.88)^***^	0.74 (0.69–0.79)^***^
N	94,454	94,454	94,454	94,454

* p < 0.05;

** p < 0.01;

*** p < 0.001.

### Demographic and clinical characteristics of suicide decedents by suicide methods

Table 3 shows that for both sexes, those age 45–64 made up the largest proportions of the decedents who died by firearm, poisoning, or other methods, and those age 25–44 made up the largest proportion of the decedents who died by hanging/suffocation. Non-Hispanic Whites were the absolute majority across all four suicide methods, but disproportionately higher shares of Hispanics and Asians/Pacific Islanders used hanging/suffocation and other methods. Those with college education comprised higher proportions of poisoning and other method users than firearms and hanging/suffocation users. Those with military service history and residents of the South were overrepresented among firearm users. More females than males had a prior suicide attempt history; however, in both sexes, those who died by poisoning included the highest proportion with such a history and those who died by firearms included the lowest. Larger proportions of those who died by poisoning and firearms disclosed their suicidal intent than those who died by hanging/suffocation and other methods. A majority of disclosers disclosed to their partner or other family members, but larger proportions of poisoning and other method users than firearm and hanging/suffocation users disclosed to healthcare providers. The data show that a higher proportion of females than males left a suicide note.

**Table 3:** Demographic and clinical characteristics of suicide decedents by sex and suicide method (column %).

***N* %**	**Men (*****N*** = **70,042; 78.4% of both sexes)**				**Women (*****N*** = **20,412; 21.6% of both sexes)**			
	**Firearm 40,502 54.7%**	**Hanging/ Suffocation 21.051 28.4%**	**Poisoning 6,664 9.0%**	**Other 5,825 7.9%**	**Firearm 6,114 30.0%**	**Hanging/ Suffocation 5,828 28.5%**	**Poisoning 6,544 32.1%**	**Other 1,926 9.4%**
Age group (%)								
18–24 years	10.9	13.0	7.3	12.8	8.2	14.0	5.5	9.6
25–44 years	28.3	45.5	30.5	35.8	31.7	44.4	26.8	33.3
45–64 years	33.2	33.3	45.2	37.0	43.3	33.3	48.6	41.8
65+ years	27.6	8.2	17.0	14.3	16.8	8.4	19.1	15.3
Race/ethnicity (%)								
Non-Hispanic White	85.3	74.1	85.2	69.8	87.3	72.5	86.1	70.9
Black/African American	6.5	6.0	4.8	10.0	5.1	6.5	5.2	9.2
Hispanic	5.1	12.4	5.4	12.0	3.9	10.2	4.5	11.3
Asian/Pacific Islander	1.3	3.9	2.8	5.4	1.7	6.8	2.3	6.4
Other	1.8	3.6	1.8	2.8	2.0	4.0	1.8	2.3
Education (%)								
High school or less	55.8	61.7	48.5	49.2	45.7	45.1	43.0	42.3
Some college/associate's degree	24.9	20.6	25.1	23.9	30.9	28.4	30.2	26.0
Bachelor's degree or higher	17.3	15.2	23.7	23.2	21.7	24.4	24.5	28.2
Unknown	2.0	2.5	2.7	3.7	1.7	2.1	2.3	3.5
Military service history (%)	26.5	10.6	17.6	12.1	4.6	2.4	2.2	1.5
Injured at home (%)	70.0	69.1	71.2	31.2	75.9	79.3	80.8	36.2
Region of residence								
Northeast	12.2	20.6	20.8	24.2	9.2	21.0	19.5	25.9
Midwest	29.0	28.2	28.6	23.9	24.9	27.4	26.9	21.4
South	31.5	23.1	21.7	19.9	38.6	21.7	24.2	21.3
West	27.0	25.7	28.5	29.2	27.2	28.4	29.1	28.7
Puerto Rico/Other (US territories/unknown)	0.3	2.4	0.5	2.9	0.1	1.5	0.3	2.7
Suicide attempt history (%)	8.9	20.5	26.0	20.1	21.0	32.3	36.5	33.6
Disclosed suicidal intent within last month (%)	21.8	20.7	23.2	19.2	22.6	20.6	23.0	19.8
Disclosed to (among those who disclosed; %)								
Previous or current intimate partner	43.1	44.3	34.6	29.2	43.0	40.0	29.2	26.0
Other family member	30.2	29.6	33.3	35.2	31.8	30.4	36.5	37.5
Friend/colleague	12.3	11.6	13.9	11.0	13.7	13.4	14.1	12.1
Neighbor	1.4	0.9	2.1	0.8	1.5	1.0	2.2	2.4
Healthcare worker	3.2	4.1	7.3	6.7	3.8	4.3	9.2	7.6
Left suicide note/electronic message (%)	27.0	26.1	42.6	20.2	34.8	32.8	43.8	26.3
Depressed mood at the time of injury (%)	30.5	32.2	30.3	24.5	32.6	34.1	30.8	25.1
Any recent mental health disorder^a^ (%)	34.5	44.7	52.1	46.3	52.0	61.2	65.9	59.7
Types of disorder (among all decedents) (%)								
Depressive disorder/dysthymia	25.8	33.7	38.6	28.7	41.1	47.9	50.9	40.2
Bipolar disorder	3.3	6.8	10.1	9.0	8.6	11.5	14.5	13.2
Anxiety disorder	6.3	7.9	11.1	7.8	12.6	14.9	18.0	10.5
Post-traumatic stress disorder	2.7	2.0	2.9	1.8	2.1	2.8	3.0	2.1
Schizophrenia	1.3	3.2	4.6	7.4	1.4	2.5	3.6	6.8
Alcohol problem/addiction (%)	16.7	20.1	21.3	14.9	14.2	16.0	17.2	11.6
Other substance misuse^b^ (%)	10.2	22.7	22.4	17.4	12.1	20.5	22.3	13.6
Other addiction^c^ (%)	0.6	0.9	0.9	0.7	0.6	0.7	0.7	0.6
Summary of mental disorder^d^ and/or SUD^e^ (%)							
No mental disorder or SUD	53.2	38.3	34.4	42.1	40.0	28.9	25.4	33.9
SUD only	12.3	17.0	13.5	11.6	8.0	9.9	8.6	6.4
Mental disorder only	23.4	26.1	29.5	30.9	37.5	41.0	41.7	45.4
Both mental disorder and SUD	11.1	18.5	22.5	15.4	14.5	20.1	24.3	14.3
Mental health/substance misuse treatment^f^ (%)								
At the time of injury	16.3	22.2	31.2	24.1	28.5	34.7	43.8	34.3
History of mental health treatment	23.2	32.0	39.7	33.9	37.8	45.8	52.7	44.8
Suicide precipitants (%)								
Physical health problem^g^	25.2	10.2	22.9	12.7	21.6	11.6	26.1	13.3
Relationship problem^h^	32.9	35.8	28.2	20.2	37.1	37.1	27.0	20.5
Death/suicide of family/friend or traumatic anniversary	8.2	7.0	7.9	5.2	10.5	9.0	10.9	6.2
Job-related or financial problem or eviction/loss of housing	16.0	18.4	16.0	13.3	14.2	14.4	11.8	10.4
Criminal or civil legal problem	9.7	13.8	10.7	8.7	5.6	8.1	5.4	4.8
Number of crises^i^, M (SE)	0.37 (0.01)	0.34 (0.01)	0.33 (0.01)	0.25 (0.01)	0.34 (0.01)	0.30 (0.01)	0.28 (0.01)	0.22 (0.01)

Within each sex, all differences by suicide methods are significant at p < 0.01 based on Pearson's χ^2^ tests except: disclosures to friend/colleagues for men (p = 0.072) and other addiction problems among women (p = 0.648). One-way ANOVA with Bonferroni corrections for the number of crises were significant at p < 0.01 for both sexes.

a Including those disorders and syndromes listed in the Diagnostic and Statistical Manual of Mental Disorders, Fifth Edition (DSM-5) with the exception of alcohol and other substance dependence.

b Including illicit drug use, even if addiction or abuse is not specifically mentioned. The exception to this is marijuana use. For marijuana, the use must be noted as chronic, abusive, or problematic.

c An addiction other than alcohol or other substance abuse (e.g., gambling, sexual) that appears to have contributed to the death.

d Including any mental health diagnosis.

e Inclusive of alcohol or other substance misuse/addiction and other addiction.

f Inclusive of pharmacotherapy, psychotherapy/counseling, any class (e.g., anger management) attendance, any facility-based care, and alcohol or narcotics anonymous.

g Including any terminal/other illness, debilitating condition, chronic/acute pain, or other physical/functional issue (perceived, or diagnosed) that were relevant to suicide.

h Problems with intimate partner and/or other family/relatives, other family stressors, caregiver burden, arguments, or abuse by a caregiver.

i Total number of contributing factors that posed a crisis within 2 weeks of death.

Women had higher proportions of those with any mental disorder (and those who received treatment) than men, with depression as the leading disorder; however, in both sexes, those who died by poisoning included the highest proportion with mental disorder/SUD, followed by those who died by hanging/suffocation. In both sexes, physical health and relationship problems were the most common precipitating factors. Physical health problems were more common in men who died by firearms followed by those who died by hanging/suffocation and in women who died by poisoning followed by those who died by firearms. Relationship problems were more common among those who died by firearms or hanging/suffocation in both sexes. The average numbers of crises were small across sex and suicide methods.

### Correlates of firearm, hanging/suffocation, and poisoning suicide in men

Table 4 shows that among men, the likelihood of firearm (vs. no firearm) use was higher in the 18–24 age group (IRR = 1.04, 95% CI = 1.01, 1.08) and the 65+ age group (IRR = 1.22, 95% CI = 1.18, 1.25), compared to the 45–64 age group. The likelihood was also higher among residents of in the South, Midwest, and West, compared to the Northeast; those with some college education; those with military service history; and those who disclosed their suicidal intent. In terms of suicide precipitants, the firearm use likelihood was higher among those with physical health problems (IRR = 1.14, 95% CI = 1.11, 1.17), relationship problems (IRR = 1.15, 95% CI = 1.12, 1.17), death/suicide of family/friend (IRR = 1.05, 95% CI = 1.01, 1.09), and more crises (IRR = 1.04, 95% CI = 1.03, 1.06).

**Table 4:** Correlates of firearm, hanging/suffocation, and poisoning use among men: Results from generalized linear modeling for a Poisson distribution with a log link.

	**Firearm vs. all other**	**Hanging/suffocation vs. all other**	**Poisoning vs. all other**
	**IRR (95% CI)**	**IRR (95% CI)**	**IRR (95% CI)**
Age group: vs. 45–64 years			
18–24 years	1.04 (1.01–1.08)^*^	1.08 (1.03–1.14)^**^	0.56 (0.50–0.61)^***^
25–44 years	0.90 (0.88–0.92)^***^	1.29 (1.25–1.33)^***^	0.71 (0.67–0.76)^***^
65+ years	1.22 (1.18–1.25)^***^	0.58 (0.55–0.62)^***^	0.69 (0.64–0.75)^***^
Race/ethnicity: vs. Non-Hispanic White			
Black/African American	0.98 (0.94–1.02)	0.90 (0.85–0.96)^**^	0.87 (0.78–0.98)^*^
Hispanic	0.74 (0.71–0.77)^***^	1.39 (1.33–1.46)^***^	0.76 (0.68–0.84)^***^
Asian/Pacific Islander	0.54 (0.49–0.58)^***^	1.58 (1.47–1.69)^***^	1.10 (0.95–1.27)
Other	0.76 (0.71–0.82)^***^	1.42 (1.32–1.53)^***^	0.80 (0.67–0.94)^*^
Region of Residence: vs. Northeast			
Midwest	1.34 (1.29–1.38)^***^	0.78 (0.75–0.82)^***^	0.81 (0.76–0.87)^***^
South	1.47 (1.43–1.52)^***^	0.70 (0.67–0.73)^***^	0.67 (0.62–0.72)^***^
West	1.37 (1.32–1.41)^***^	0.71 (0.68–0.74)^***^	0.84 (0.78–0.90)^***^
Puerto Rico/Other	0.53 (0.44–0.63)^***^	1.22 (1.11–1.35)^***^	0.40 (0.28–0.57)^***^
Education: vs. High school or less			
Some college/associates' degree	1.05 (1.03–1.08)^***^	0.81(0.78–0.84)^***^	1.22 (1.15–1.30)^***^
Bachelor's degree or higher	0.95 (0.93–0.98)^***^	0.86 (0.82–0.89)^***^	1.43 (1.34–1.52)^***^
Unknown	0.86 (0.80–0.92)^***^	1.03 (0.94–1.12)	1.36 (1.17–1.58)^***^
Military service history	1.19 (1.16–1.22)^***^	0.68 (0.65–0.71)^***^	0.88 (0.82–0.94)^***^
Suicide attempt history	0.64 (0.62–0.66)^***^	1.29 (1.24–1.33)^***^	1.84 (1.73–1.95)^***^
Disclosed suicide intent	1.03 (1.01–1.06)^*^	0.91 (0.88–0.95)^***^	1.07 (1.01–1.13)^*^
Depressed mood at the time of injury	1.01 (0.98–1.03)	1.08 (1.05–1.12)^***^	0.90 (0.85–0.95)^***^
Mental disorder/SUD: vs. no disorder			
SUD only	0.85 (0.82–0.87)^***^	1.25 (1.20–1.30)^***^	1.35 (1.25–1.46)^***^
Mental disorder(s) only	0.86 (0.84–0.88)^***^	1.19 (1.15–1.23)^***^	1.33 (1.25–1.41)^***^
Both mental disorder and SUD	0.76 (0.74–0.79)^***^	1.26 (1.21–1.32)^***^	1.77 (1.65–1.90)^***^
Physical health problem	1.14 (1.11–1.17)^***^	0.67 (0.64–0.70)^***^	1.22 (1.15–1.30)^***^
Relationship problem	1.15 (1.12–1.17)^***^	0.99 (0.96–1.02)	0.81 (0.76–0.86)^***^
Death/suicide of family/friend	1.05 (1.01–1.09)^**^	0.96 (0.91–1.01)	1.01 (0.92–1.11)
Criminal or civil legal problem	0.91 (0.88–0.94)^***^	1.19 (1.14–1.24)^***^	1.04 (0.96–1.13)
Job-related, financial, or housing problem	1.00 (0.97–1.03)	1.10 (1.06–1.14)^***^	0.87 (0.81–0.93)^***^
No. of crises	1.04 (1.03–1.06)^***^	0.94 (0.91–0.96)^***^	0.96 (0.92–1.01)
N	74,042	74,042	74,042

* p < 0.05;

** p < 0.01;

*** p < 0.001.

The likelihood of hanging/suffocation (vs. no hanging/suffocation) was higher in the 18-24 age group (IRR = 1.08, 95% CI = 1.03, 1.14) and the 25–44 age group (IRR = 1.29, 95% CI = 1.25, 1.33), compared to the 45-64 age group, and among Hispanic, Asian/Pacific Islander, and “other” racial/ethnic groups, compared to non-Hispanic Whites. The likelihood was also higher among those with any suicide attempt history (IRR = 1.29, 95% CI = 1.24–1.33); depressed mood at the time of injury (IRR = 1.08, 95% CI = 1.05–1.12); any mental disorder/SUD; any legal problem (IRR = 1.19, 95% CI = 1.14, 1.24); and job/financial/housing problem (IRR = 1.10, 95% CI = 1.06, 1.14).

The likelihood of poisoning (vs. no poisoning) was significantly lower in the other three age groups compared to the 45–64 age group but higher among those with at least some college education and those who disclosed their suicide intent. Other correlates of poisoning were suicide attempt history (IRR = 1.84, 95% CI = 1.73–1.95); any mental health/SUD; and physical health problems (IRR = 1.22, 95% CI = 1.15, 1.30). These findings largely support our hypothesis.

### Correlates of firearm, hanging/suffocation, and poisoning suicide in women

Table 5 shows that among women, the likelihood of firearm use was lower among the two younger groups compared to the 45–64 age group and among racial/ethnic minorities compared to non-Hispanic Whites; but higher among residents of the South, Midwest, and West, compared to those of the Northeast. The likelihood of firearm use was lower among those with mental disorder/SUD but higher among those with relationship problems and more crises.

**Table 5:** Correlates of firearm, hanging/suffocation, and poisoning use among women: Results from generalized linear modeling for a Poisson distribution with a log link.

	**Firearm**	**Hanging/suffocation**	**Poisoning**
	**IRR (95% CI)**	**IRR (95% CI)**	**IRR (95% CI)**
Age group: vs. 45–64 years			
18–24 years	0.89 (0.81–0.98)^*^	1.74 (1.59–1.89)^***^	0.59 (0.53–0.66)^***^
25–44 years	0.93 (0.87–0.99)^*^	1.54 (1.45–1.63)^***^	0.72 (0.68–0.76)^***^
65+ years	1.03 (0.95–1.11)	0.78 (0.70–0.86)^***^	1.10 (1.03–1.18)^**^
Race/ethnicity: vs. Non-Hispanic White			
Black/African American	0.75 (0.67–0.84)^***^	1.08 (0.97–1.20)	1.01 (0.90–1.12)
Hispanic	0.62 (0.55–0.71)^***^	1.39 (1.27–1.53)^***^	0.78 (0.69–0.88)^***^
Asian/Pacific Islander	0.43(0.35–0.52)^***^	1.83 (1.65–2.04)^***^	0.65 (0.55–0.77)^***^
Other	0.73(0.61–0.87)^**^	1.47 (1.28–1.68)^***^	0.80 (0.66–0.96)^*^
Region of residence: vs. Northeast			
Midwest	1.76 (1.60–1.94)^***^	0.87 (0.81–0.94)^***^	0.93 (0.86–0.99)^*^
South	2.50 (2.28–2.74)^***^	0.68 (0.63–0.73)^***^	0.79 (0.73–0.85)^***^
West	1.87 (1.70–2.07)^***^	0.79 (0.73–0.85)^***^	0.92 (0.85–0.99)^*^
Puerto Rico/Other	0.28 (0.11–0.67)^**^	1.24 (0.99–1.55)	0.38 (0.23–0.60)^***^
Education: vs. High school or lower			
Some college/associates' degree	1.01 (0.96–1.08)	0.92 (0.86–0.97)^**^	1.08 (1.02–1.14)^*^
Bachelor's degree or higher	0.90 (0.84–0.96)^**^	1.02 (0.95–1.09)	1.03 (0.97–1.10)
Unknown	0.79 (0.65–0.95)^*^	0.99 (0.82–1.19)	1.06 (0.90–1.25)
Suicide attempt history	0.66 (0.62–0.71)^***^	1.03 (0.97–1.09)	1.27 (1.20–1.33)^***^
Disclosed suicide intent	1.04 (0.98–1.11)	0.88 (0.83–0.94)	1.06 (1.00–1.12)
Depressed mood at the time of injury	1.05 (0.99–1.11)	1.13 (1.07–1.20)^***^	0.91 (0.86–0.96)^**^
Mental disorder/SUD: vs. no disorder			
SUD only	0.71 (0.64–0.78)^***^	1.17 (1.06–1.29)^**^	1.34 (1.22–1.48)^***^
Mental disorder(s) only	0.80 (0.75–0.85)^***^	1.11 (1.04–1.18)^**^	1.19 (1.11–1.26)^***^
Both mental disorder and SUD	0.63 (0.58–0.69)^***^	1.07 (0.99–1.16)	1.56 (1.44–1.67)^***^
Physical health problem	1.06 (0.99–1.13)	0.69(0.64–0.75)^***^	1.29 (1.22–1.37)^***^
Relationship problem	1.30 (1.23–1.38)^***^	1.07 (1.01–1.13)^*^	0.83 (0.78–0.88)^***^
Death/suicide of family/friend	1.06 (0.97–1.15)	0.97 (0.88–1.06)	1.07 (0.99–1.16)
Criminal or civil legal problem	0.83 (0.74–0.93)^**^	1.21 (1.09–1.33)^***^	0.97 (0.86–1.08)
Job-related, financial, or housing problem	1.07 (0.99–1.16)	1.11 (1.03–1.19)^**^	0.88 (0.81–0.95)^**^
No. of crises	1.07 (1.02–1.11)^**^	0.95 (0.91–0.99)^*^	0.98 (0.94–1.03)
_N	20,412	*N* = 20,412	20,412

Due to a small proportion of cases with military service history, the variable was not included in the model.

* p < 0.05;

** p < 0.01;

*** p < 0.001.

The likelihood of hanging/suffocation was higher in the 18–24 age group (IRR = 1.74, 95% CI = 1.59, 1.89) and the 25–44 age group (IRR = 1.54, 95% CI = 1.45, 1.63), compared to the 45–64 age group, and Hispanic, Asian/Pacific Islander, and “other” race/ethnic groups, compared to non-Hispanic Whites. The likelihood was also higher among those with depressed mood at the time of injury (IRR = 1.13, 95% CI = 1.07–1.20); mental disorder or SUD; relationship problem (IRR = 1.07, 95% CI = 1.01, 1.13); any legal problem (IRR = 1.21, 95% CI = 1.09, 1.33); and job/financial/housing problem (IRR = 1.11, 95% CI = 1.03, 1.19).

The likelihood of poisoning was lower in two younger age groups but higher in the 65+ age group (IRR = 1.10, 95% CI = 1.03–1.18), compared to the 45–64 age group. Other correlates of poisoning use were some college education; suicide attempt history (IRR = 1.27, 95% CI = 1.20–1.33); any mental disorder/SUD; and physical health problems (IRR = 1.29, 95% CI = 1.22, 1.37). Again, these findings largely support our hypotheses.

## Discussion

In this paper, we used the 2017-2019 NVDRS to examine demographic and clinical correlates of three leading methods of suicide—firearms, hanging/suffocation, and poisoning—among decedents age 18 and older. Consistent with the CDC report (3), our findings show that firearm use increased and poisoning use decreased during the study period. While changes in hanging/suffocation during the 3 years were not statistically significant, it was used by almost 30% of both male and female decedents. Among female decedents, poisoning (32.1%) was still a bit more frequent than firearms (30.0%) during the study period, although CDC reported that firearms became the leading method among female decedents in 2020 (3).

Increased firearm use even during the three-year study period is not surprising given that the numbers of manufactured or imported firearms have increased continuously over the past three decades (e.g., 11.5 million firearms manufactured in 2016 alone) (19), and that nearly 28 million firearms were sold in 2018 and 2019 (20). In 2020, firearm sales surged to 23 million (21), which also means increased exposure and easy access to lethal methods among the purchasers' household members (22). Studies of recent firearm sales also showed that new firearm owners were twice more likely than those who did not own firearms to report lifetime, past-year, and past-month suicidal ideation and that half of new owners were women (22, 23).

Our multivariable analyses show that male decedents age under 45 had higher likelihood of firearm and/or hanging/suffocation use than those age 45–64. Female decedents age under 45 also had higher likelihood of hanging/suffocation use than those age 45–64. High rates of firearm use among the 65+ age group have been well documented, and attributed to a greater intent to die and greater premeditation (7, 9, 24, 25). However, the higher likelihood of using firearms and/or hanging/suffocation, two highly lethal methods, among decedents age under 45 than those age 45–64 is concerning. We speculate that some suicides in the under-45 age group may have been impulsive and not premeditated (e.g., following arguments related to relationship conflicts) (26, 27). We need to develop means restriction approaches that target firearms and hanging/suffocation for all age groups, and those approaches may have more impact on young and middle-aged people than older people.

In addition to age group differences, racial/ethnic and geographic variations in suicide methods show that method choice is influenced by culture/norms of the racial/ethnic group and familiarity with and high acceptability of the methods, especially firearms, fostered by their easy availability/accessibility. The finding that women in the South [where gun ownership is high (28)] were 2.5 times more likely than women in the Northeast to die by firearms is consistent with the previous findings of positive relationships between household/self gun ownership and firearm suicide (28–33). Hispanics and Asian/Pacific Islanders were significantly less likely than Non-Hispanic White to use firearms but they were more likely to use hanging/suffocation. This may be a reflection of the overall lower gun ownership rates among Hispanics and Asian/Pacific Islanders compared to non-Hispanic Whites (34). However, firearm retailer surveys in 2020 and 2021 showed sharp increase in gun purchases among African American, Hispanic, and Asian Americans (35).

Consistent with the findings of Jamison and Bol's study (8), prior suicide attempt history was associated with higher likelihood of poisoning use in both sexes. Male decedents with prior suicide attempt history in our study were also more likely to die by hanging/suffocation. However, it is not clear if this was due to their restricted access to firearms (e.g., family members took them away) since they disclosed their suicidal intent or attempted suicide previously. Hanging/suffocation or poisoning may have been used as a substitution of a firearm for some decedents who had disclosed their suicide intent, even though individual-level substitution of methods is difficult to assess (36, 37). While means restriction at the population level has strong empirical support as a suicide prevention strategy (38, 39), individuals with strong intention to die could have used the next lethal method, hanging/suffocation or poisoning, when they could not access firearms. Reasons for positive associations between intent disclosure and use of hanging/suffocation and poisoning but not firearms need further examination.

Mental and substance use disorders were associated with higher likelihood of hanging/suffocation and poisoning in both sexes. Physical health problems were also associated with higher likelihood of poisoning in both sexes and higher likelihood of firearm use in men. The latter is likely because three quarters of male decedents age 65+ used firearms and they were most likely to have had physical health problems as a suicide precipitant. A previous NVDRS-based study found that physical health problems were recorded as a suicide precipitant for 50% of suicide decedents age 65+, compared to 23% and 12% of decedents age 50-64 and age 30-49, respectively, and that among the 65+ age group, male decedents and those age 85+ had significantly higher rates of precipitating physical health problems (40).

Relationship problems were associated with higher likelihood of firearm use in both sexes and higher likelihood of hanging/suffocation in women. A systematic review found that relationship discord is a significant risk factor for suicidal thoughts and suicide attempt (41). Some of those with relationship problems (or other types of crises) who used poisoning may have survived, but not those who used firearms or hanging/suffocation, even if such problems are risk factors for an impulsive suicidal act.

Criminal/civil legal problems and job/financial/housing problems were associated with higher likelihood of hanging/suffocation in both sexes, which is not surprising as these problems were likely to be more prevalent among young/middle-aged decedents (42) who had high rates of hanging/suffocation use. A previous NVDRS-based study showed that county-level poverty rates and foreclosure rates were strongly associated with suicide rates for both genders and all age groups, but the association was stronger for the 45–64 age group, and poverty rates were also associated with increased alcohol involvement for male decedents aged 45–64 years (43). Other studies also found that job insecurity, other job stressors, and economic insecurity/hardship in young and middle-aged adults are associated with increased psychological distress and suicidal ideation (44, 45). A previous epidemiologic, longitudinal study also found that US adults with debt, housing instability, unemployment, and low income at wave 1 had 20 times higher predicted probability of attempting suicide compared with respondents endorsing none of these variables at wave 2 (2–3 years later) (46). More often than not, economic hardship is accompanied by physical (e.g., chronic illnesses and pain) and mental health problems and substance misuse in middle-aged individuals, especially non-Hispanic Whites, resulting in increased mortality from suicide, drug overdose, and chronic illnesses over the past decades (47). Our finding that more crises were associated with higher likelihood of firearm use in both sexes also suggests that decedents who faced multiple stressors likely had stronger intention to die.

This study had limitations. First, NVDRS data on physical and mental health problems and substance misuse were collected from decedents' family/friends and other informants and/or suicide notes, not from healthcare providers. Without diagnosis data and input from healthcare providers, the validity of these data could not be ascertained. NVDRS allowed that contributing health problems may have been perceived and psychosomatic (influenced by depression and/or other affective or cognitive disorders) rather than formally diagnosed. Second, although a majority of states participated in the 2017-2019 NVDRS, some states did not provide data on all 3 years and others provided only partial data limited to some counties. Thus, the findings are not representative of all US older-adult suicide decedents. Third, while CDC data showed that rural male and female hanging/suffocation suicide rates more than doubled and quadrupled, respectively, between 2000 and 2018 (2), we did not examine rural vs. urban differences in this study for lack of data. Future research needs to be more granular about geographic differences.

Given the serious public health crises related to high rates of suicide attempts and deaths in the U.S., our findings, despite the above data limitations, have some important clinical and policy implications for suicide prevention. First, increasing use of lethal methods among young and middle-aged people indicates the urgent need for reducing access to these methods. While examples from other countries showed that legislation reducing firearm ownership lowers firearm suicide rates, the Second Amendment in the U.S. curtails legislation broadly restricting firearm access (48). In the District of Columbia v Heller (2008) and McDonald v City of Chicago (2010) cases, the Supreme Court held that the Second Amendment grants individuals a personal right to possess handguns in their home for protection (49). However, a panel of state level data for the years 1995–2004 in the US showed that gun control measures such as permit and licensing requirements had a negative effect on male suicide rates (50). A systematic review also showed that laws that strengthen background checks and permit-to-purchase seemed to decrease firearm homicide rates, but it also noted poor quality data and weak study designs (51). For firearm owners [most suicides involve guns purchased years earlier (48)], safety strategies, which emphasize the reduction of practical capability for suicide through the limitation of access to and safe storage of firearms, along with counseling for psychosocial risk factors, have been found somewhat effective (52–54). Especially for young people whose suicidal acts likely reflect multiple facets of impulsivity (26), access restrictions likely help reduce easy access to firearms during times of crisis.

Second, given many different ways from which asphyxiation can be derived, there are no practical ways to restrict hanging/suffocation. Thus, the best way to reduce its use is to provide needed help for reducing their suicidal thoughts and behaviors. Significance of prior suicide attempt history and mental disorder/SUD as correlates of higher likelihood of hanging/suffocation and poisoning indicates the urgency of broadening mental health/substance use treatment accessibility for at-risk individuals in order to provide psychosocial support and better coping skills training for negative life events and adversities. A study found that more than 30% of the U.S. adults who made suicide plans and/or attempted suicide received no treatment before or after planning or attempting (55). Cost concerns and lack of insurance were the most frequently mentioned reasons for not seeking mental health and/or substance use treatment among young and middle-aged individuals (56). The majority of young people who are struggling with suicidal thoughts seek help from social networks that most commonly consist of peers (57). Thus, these informal support systems for at-risk individuals also need to be informed about and trained in helping them access treatment programs. Suicide prevention training programs for informal support systems such as ASIST (Applied Suicide Intervention Skills Training) have been found effective (58, 59).

Third, physical health problems as a significant correlate of suicide underscore the importance of improved long-term and palliative care services so that those (mostly older adults) with terminal illnesses and bodily pain can be supported without feeling burdensome to their families. A systematic review of reviews found that physical activity and collaborative management at integrated physical and behavioral health settings to be effective for reducing suicidal behaviors among older adults (60). It is also important to provide support for older adults' informal caregivers to reduce their caregiving burden and to train them in practical tools and skill sets for detecting suicidal ideation/intent in their loved ones and implementing suicide prevention steps.

Fourth, the association between job/financial/housing problems and hanging/suffocation among young and middle-aged adults also points to the importance of strengthening safety net programs and bridging support to alleviate financial hardship and housing difficulties, along with physical and mental health services, for people who are experiencing job/financial/housing crises. For those living “lives of despair” (61) and hopelessness from combined forces of economic insecurity, pending eviction, and physical and psychiatric illnesses, mental health support alone is not likely to be enough to alleviate their sufferings. Interventions at systemic levels, including financial/housing support and physical/mental healthcare policies (e.g., generous unemployment and health insurance, housing assistance, easy access to legal aid services) that are designed to mitigate both economic and health disparities among underserved and vulnerable sectors of the population are needed. Recent studies have shown significant associations between increased state minimum wage and decreased suicide rates (62, 63).

Fifth, more research is also needed to identify effective strategies to curtail the increasing use of firearm and hanging/suffocation among young and middle-aged adults. For those at risk of poisoning suicide, healthcare providers also need to monitor their medication and other substance use carefully and other risks of poisoning.

In conclusion, firearms, hanging/suffocation, and poisoning accounted for a majority of U.S. adult suicides in 2017–2019. While high rates of firearm use among the 65+ age group have been well-documented, high rates of firearm and hanging/suffocation use among young and middle-aged groups (those age under 45) are troubling. Our findings of suicide contributors associated with each method point to the need for more effective suicide prevention strategies that include means restriction, mental health/substance use treatment, alleviation of economic hardship, and improved long-term and palliative care services for older adults with physical health problems.

## Data availability statement

The data analyzed in this study is subject to the following licenses/restrictions: The authors were granted access to the NVDRS-RAD (Restricted Data Access) based on the NVDRS-RAD review committee's review of our proposal. The authors are not allowed to share the data set with unauthorized people. Requests to access these datasets should be directed to nvdrs-rad@cdc.gov.

## Ethics statement

This study based on de-identified decedents was exempt from the authors' Institutional Review Board's review.

## Author contributions

NC and BC conceptualized and conducted literature review. NC did data analysis and drafted the paper. CM provided statistical consultation. All authors made substantial contributions, approved the final version, and edited the paper.

## Funding

The authors received support from the University of Texas at Austin's internal research fund.

## Conflict of interest

The authors declare that the research was conducted in the absence of any commercial or financial relationships that could be construed as a potential conflict of interest.

## Publisher's note

All claims expressed in this article are solely those of the authors and do not necessarily represent those of their affiliated organizations, or those of the publisher, the editors and the reviewers. Any product that may be evaluated in this article, or claim that may be made by its manufacturer, is not guaranteed or endorsed by the publisher.
